# Clinical research, mechanisms, and prospects of flavonoids from Herba *Patriniae* in the treatment of colorectal cancer

**DOI:** 10.3389/fphar.2025.1633286

**Published:** 2025-08-28

**Authors:** PingPing Zhang, Ru Jia, Yao Wang, YuFei Tang, Qi Li, FengGang Hou

**Affiliations:** ^1^ Department of Medical Oncology, Shanghai Municipal Hospital of Traditional Chinese Medicine, Shanghai University of Traditional Chinese Medicine, Shanghai, China; ^2^ Department of Medical Oncology, Shuguang Hospital, Shanghai University of Traditional Chinese Medicine, Shanghai, China; ^3^ Department of Chinese Medicine and Integrative Medicine, Shanghai Geriatric Medical Center, Zhongshan Hospital, Fudan University, Shanghai, China

**Keywords:** Herba *Patriniae*, flavonoids, colorectal cancer, gut microbiota, anticancer

## Abstract

Colorectal cancer (CRC) is a common and aggressive malignancy of the gastrointestinal tract with a severe disease burden. The role of Traditional Chinese Medicine (TCM) and its natural active ingredients in enhancing the therapeutic effects of radiotherapy and chemotherapy and preventing the recurrence and metastasis of CRC has been increasingly recognized. Herba *Patriniae* has shown significant clinical efficacy for the treatment of CRC. Flavonoids has been found to be one of the main active anticancer components of Herba *Patriniae*. This review summarizes the latest findings from clinical trials and *in vitro* studies on anticancer mechanisms of Herba *Patriniae*, and discusses the role of the flavonoids in combination therapy against CRC. These flavonoids exert anticancer effects through diverse mechanisms. For instance, they prevent the development of precancerous lesions, regulate the cell cycle, modulate CRC cell proliferation, promote tumor cell apoptosis, inhibite epithelial-mesenchymal transition, reverse drug resistance, and modulate gut microbiota by acting on several key signaling pathways, such as PI3K/Akt/mTOR, Wnt/β-catenin, and EGFR/ERK/MAPK. Future research should prioritize clarifying the specific dosage and safety of flavonoids under different pathological conditions, further conducting large-scale, rigorously designed clinical studies to determine the efficacy differences of flavonoids for patients with different pathological types of CRC and simultaneously delving into the mechanisms of their anti-colorectal cancer effects, as well as their interactions with the intestinal microbiota and tumor microenvironment.

## Introduction

Based on the latest data from the International Agency for Research on Cancer (IARC), new cases of CRC accounted for 10.2% of all incident malignant neoplasms, making it the third most common cancer worldwide. CRC-associated death accounts for 9.3% of all malignant neoplasms, making it the second deadliest cancer after lung cancer (Bray et al., 2024; Global Nutrition Target Collaborators, 2025). Due to the influence of dietary habits, lifestyle and economic development (Vernia et al., 2021), CRC is becoming more prevalent among younger people. The overall burden of the disease is still increasing, particularly in developing countries (Morgan et al., 2023), this presents a major challenge for the clinical management of CRC. Genetically, colorectal cancer (CRC) can be genetically divided into two main types: hereditary and sporadic. Hereditary CRC primarily includes familial adenomatous polyposis (FAP) and Lynch syndrome. Sporadic CRC, which accounts for approximately 75% of cases, is mainly caused by gene mutations triggered by environmental factors. The occurrence and progression of sporadic CRC frequently involve the inactivation of genes such as APC, DCC, and TP53, over-expression of mutations in genes like KRAS, PIK3CA, and BRAF, as well as deletions in DNA mismatch repair genes. Most cases of CRC cases follow the “adenoma-cancer” sequence of progression, it takes between 5 and 10 years for an adenoma to advance to cancer (Zhu et al., 2020; Sun et al., 2024). Treatment for CRC mainly includes surgical resection, alongside and radiotherapy. However, 25%–30% of patients have metastasis when their initial diagnosis, and 50% of patients experience recurrent metastasis within 5 years of surgery (Ciardiello et al., 2022). Patients diagnosed with advanced CRC face many limitations, such as resistance to chemotherapy and serious adverse reactions. In-depth studies on molecular subtypes have greatly improved the 5-year survival rate of patients receiving molecularly targeted drugs (Ciardiello et al., 2022), such as drugs targeting vascular endothelial growth factor/vascular endothelial growth factor receptor (VEGF/VEGFR) and epidermal growth factor receptor (EGFR) (Xie et al., 2020). Such studies have also improved the prognosis of patients by detecting their microsatellite instability/mismatch repair (MSI/MMR) and programmed death ligand 1 (PD-L1) status and adding immunotherapy (André et al., 2025). Current pharmacological interventions for CRC primarily focus on single-target agents; however, the therapeutic efficacy of drugs inhibiting individual signaling pathways or biological targets remains limited. In contrast, Traditional Chinese Medicine (TCM) and its bioactive components exhibit distinct advantages through their multi-target, multi-pathway, and multi-effect regulatory mechanisms (Chen et al., 2023; Gou et al., 2023). These compounds can markedly alleviate clinical symptoms, improve quality of life, and stabilize lesions in patients with CRC. In addition, when used in combination therapies, these compounds can enhance treatment outcomes and minimize adverse effects.

Herba *Patriniae*, a wild plant with recognized medicinal and dietary value, has been utilized for over 2,000 years. It is derived from *Patrinia scabiosaefolia* or *Patrinia villosa* of the Valerianaceae family, with its rhizomes, roots, and entire herba employed for therapeutic purposes. Widely distributed across tropical and subtropical regions, Herba *Patriniae* is rich in bioactive compounds, such as triterpenes, saponins, iridoids, and flavonoids; remarkably, *Patrinia villosa* contains more flavonoids (Gong et al., 2021). These constituents contribute to its diverse pharmacological properties, including antioxidant, anti-tumor, anti-inflammatory, antimicrobial, and antiviral activities, making it a promising candidate for drug development (Li et al., 2018; Huang et al., 2019; Zou et al., 2021; Liu et al., 2023a; Li K. et al., 2024).

Recently, many studies have supported the efficacy of Herba *Patriniae* in preventing and treating CRC, although the underlying mechanisms of action remain unclear. The anticancer compounds of this plant have shown significant interactions with target proteins involved in the progression of CRC (Qi et al., 2020). Studies have suggested that Herba *Patriniae* acts through several signaling pathways to inhibit CRC, with its high flavonoid content playing a pivotal role. These flavonoids suppress cell proliferation (Zhang M. et al., 2015; Yang et al., 2023), induce apoptosis (Liu et al., 2013), mediate cell cycle arrest (Zhang M. et al., 2015), suppress angiogenesis in the tumor microenvironment (Chen et al., 2013), and ameliorate drug resistance (Huang et al., 2019). Despite their potent bioactivity, flavonoids, as polyphenolic compounds, generally have poor oral bioavailability, with only minimal metabolites detectable in urine and blood. Their interaction with gut microbiota is a critical mechanism underlying their biological activity (Liu et al., 2023b). Building on these findings, we reviewed the findings of clinical and preclinical studies focused on effective flavonoids in Herba *Patriniae* and unraveled their molecular mechanisms in CRC. This review provides a foundation for the development of anticancer small molecules derived from Herba *Patriniae* and help identify their potential therapeutic targets.

## Flavonoids in Herba *Patriniae*

Flavonoids, a class of polyphenolic compounds, have a chemical structure composed of 15 carbon atoms, including two benzene rings (A ring and B ring) and an oxygen-containing ring (C ring). The basic structure of flavonoids is generally C_15_H_10_O_2_, although their molecular formula vary based on their substituent groups. Structural diversity among flavonoids arises from substitutions on the A, B, or C rings, such as amino, methyl, hydroxyl, or glycosyl groups, resulting in derivatives with distinct biological activities (Zhang et al., 2007; Lotito et al., 2011). With a deeper understanding of the role of gut microbiota, studies have shown that flavonoid metabolism in the body relies not only on liver enzyme systems but also on gut microbiota. Through processes such as hydrolysis, reduction, and metabolic transformation, gut microbiota modifies the structure of flavonoids, enhances their biological activity, and affects their bioavailability and pharmacological properties (Kumar Singh et al., 2019; Landete, 2022). Epidemiological studies have indicated that higher flavonoid intake and serum levels are associated with a lower risk of colorectal inflammation and CRC (Chang et al., 2018). Based on an analysis of the TCM pharmacology database, we identified 13 flavonoid compounds from 52 active ingredients in Herba *Patriniae* that have been widely studied for their anticancer properties (Table 1, https://www.tcmsp-e.com/). A comprehensive understanding of the pharmacological effects of these flavonoids on CRC is critical for advancing therapeutic strategies.

**TABLE 1:** The main flavonoids of Herba *Patriniae*.

Mol ID	Molecule name	Molecular formula	Pubchem CID	OB(%)	Caco-2	HL	Structure
MOL001678	Bolusanthol B	C_20_H_20_O_6_	10594416	39.94	0.29	15.05	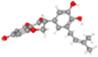
MOL001685	Orotinin	C_25_H_26_O_6_	21721831	1.01	0.64	—	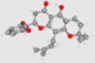
MOL001790	Linarin	C_28_H_32_O_14_	5317025	39.84	−1.68	16.07	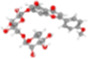
MOL001689	Acacetin	C_16_H_12_O_5_	5280442	34.97	0.67	17.25	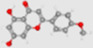
MOL002322	Isovitexin	C_21_H_20_O_10_	162350	31.29	−1.24	16.45	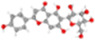
MOL001695	Quercimeritrin	C_21_H_20_O_12_	5282160	2.85	−1.36	—	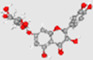
MOL001696	Morusin	C_25_H_24_O_6_	5281671	11.52	0.51	—	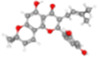
MOL000415	Rutin	C_27_H_30_O_16_	5280805	3.2	−1.93	—	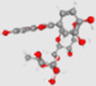
MOL000422	Kaempferol	C_15_H_10_O_6_	5280863	41.88	0.26	14.74	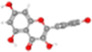
MOL000006	Luteolin	C_15_H_10_O_6_	5280445	36.16	0.19	15.94	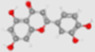
MOL000498	Isoorientin	C_21_H_20_O_11_	114776	23.3	−1.35	—	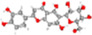
MOL000008	Apigenin	C_15_H_10_O_5_	5280443	23.06	0.43	—	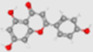
MOL000098	Quercetin	C_15_H_10_O_7_	5280343	46.43	0.05	14.40	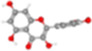

## Preclinical studies on the anticancer effects of Herba *Patriniae* and flavonoids against CRC

Crypts are deep grooves formed by epithelial cells in the colon. Abnormal crypt foci (ACF) appear when these crypts undergo significant morphological changes, such as hyperplasia, irregular shapes, or abnormal cell differentiation. ACF are one of the earliest precancerous lesions in CRC. Early detection and effective intervention can significantly reduce the risk of CRC.

Flavonoid compounds can effectively prevent ACF. For example, the number of abnormal crypts in the colons of stressed mice was significantly reduced after treatment with quercetin (Ritter and Dinh, 1992; Matsukawa et al., 1997; Volate et al., 2005). Interestingly, in an obesity-related carcinogenesis model, quercetin significantly inhibited precancerous lesions and reduced serum leptin levels. Furthermore, *in vivo* studies revealed that quercetin markedly suppressed the expression of leptin mRNA in differentiated 3T3-L1 mouse adipocytes. These findings suggest that quercetin has the potential to inhibit colorectal cancer induced by obesity (Miyamoto et al., 2010). In the mice model of AOM-induced CRC, a 0.5% quercetin diet effectively inhibited intestinal lesions (Tutino et al., 2018). Similarly, a diet containing 0.1% apigenin reduced the number of high-magnitude ACF (defined as > 4 abnormal crypts per focus) by 57% (*P* < 0.05) (Au et al., 2006; Leonardi et al., 2010). Luteolin (1.2 mg/kg/day) mitigated AOM-induced intestinal oxidative damage by reducing lipid peroxidation, enhancing antioxidant defenses, and suppressing the formation of precancerous lesions (Ashokkumar and Sudhandiran, 2008). In the APC^Min/+^ mouse model, apigenin dose-dependent inhibited tumor growth by phosphorylating the p53 protein in tumor tissue, thereby regulating tumorigenesis (Zhong et al., 2010). Treatment with kaempferol reduced tumor burden, restored impaired intestinal barriers, and downregulated the expression of Ki67 and LGR5 in APC^Min/+^ mouse model (Li X. et al., 2022). However, rutin exhibited no preventive effect in the AOM-induced mouse model, likely due to its low intestinal bioavailability. Improving the solubility of rutin using solid dispersion technology and formulating it into frankincense-based compression-coated tablets enhanced its efficacy in inhibiting the development of CRC *in vivo* (Ismail et al., 2023).

Chronic inflammation is a significant risk factor for CRC, as it promotes cancer cell proliferation, invasion, and metastasis by modulating the tumor microenvironment, activating oncogenes, immune evasion, and dysbiosis (Yaeger et al., 2016; Chen et al., 2024). Apigenin has been shown to effectively inhibit inflammatory bowel disease (IBD) and colitis-associated cancer (CAC) in mice (Ai et al., 2017). Similarly, vitexin mitigated AOM/DSS-induced chronic colitis-associated carcinogenesis in mice (Chen et al., 2021). Linarin (25 or 50 mg/kg/day) significantly reduced myeloperoxidase activity in the colon and downregulated pro-inflammatory cytokines, such as TNF-α and IL-1β, while upregulating the anti-inflammatory cytokine IL-10. This effect alleviated DSS-induced intestinal damage and the function of the mucosal and intestinal barrier (Jin et al., 2022). In a TNBS-induced experimental colitis model in Wistar rats, morusin (12.5 mg/kg) showed comparable efficacy to sulfasalazine (50 mg/kg) in suppressing inflammation (Vochyánová et al., 2017).

In a CRC metastasis models, quercetin (Kee et al., 2016) and luteolin (10 or 50 mg/kg) significantly reduced the number and volume of lung metastases induced by CT26 cells (Kim H. Y. et al., 2013). Moreover, luteolin (IC50 = 5.9 μM, indicating relatively low anticancer activity) suppressed CT26-induced liver metastasis of colon cancer by 24% (Naso et al., 2016).

## Clinical studies on the anticancer effects of flavonoids against CRC

A prospective cohort study involving 87 patients, consisting of 36 patients who underwent CRC surgery and 51 patients who underwent adenoma polypectomy, investigated the preventive effects of a flavonoid mixture (20 mg apigenin and 20 mg epigallocatechin gallate) on tumor recurrence. After 3–4 years of colonoscopy follow-up, the group receiving the flavonoid mixture exhibited a tumor recurrence rate of 7% (1/14, 1 adenoma) compared to a 47% recurrence rate (7/15, including 3 tumor recurrence and 4 adenomas) in a matched untreated control group of patients with CRC (n = 15). These findings suggest that long-term treatment with flavonoids can reduce the recurrence rate of colorectal tumors in patients undergoing surgery (Hoensch et al., 2008). A 4-year nutritional intervention trial featuring a low-fat, high-fiber, high-fruit, and high-vegetable diet found that the intake of flavonoids, such as isoquercitrin, kaempferol, and quercetin, was associated with decreased serum levels of IL-6 and a lower recurrence rate of high-risk adenomas. This finding suggests a potential correlation between flavonoid intake and a decreased risk of adenoma recurrence (Bobe et al., 2010).

Patients with familial adenomatous polyposis (FAP) suffer from a high risk of CRC. In a small clinical study, 6 months of combined treatment with curcumin and quercetin after CRC surgery led to a 60% reduction in the number and size of polyps in the ileum and rectum compared to baseline, indicating that the potential of this combination therapy in reducing polyp burden in patient with FAP (Cruz-Correa et al., 2006).

A Phase I clinical study on quercetin’s resistance and pharmacokinetics revealed the safety of intravenous quercetin. At a specific plasma concentration, it inhibited the activity of lymphocyte tyrosine kinase, showing anti-tumor effects. In nine patients with CRC, treatment with intravenous quercetin for 1 hour suppressed the phosphorylation of serum lymphocyte protein tyrosine for 16 h. Additionally, in a patient with cisplatin-resistant ovarian cancer, treatment with quercetin (420 mg/m^2^) reduced CA125 levels from 295 units/mL to 55 units/mL. Similarly, another patient with liver cancer showed decreased serum alpha-fetoprotein levels after treatment (Ferry et al., 1996).

However, a large retrospective clinical study with 38,408 participants assessed flavonoid intake using dietary questionnaires and found no significant association between the intake of five common flavonoids (quercetin, kaempferol, myricetin, apigenin, and luteolin) or flavonoid-rich foods and CRC prevention (Wang et al., 2009). This lack of correlation may be attributed to the low flavonoid content in typical diets, antibiotic use, poor dietary habits, etc.

Although Herba *Patriniae* is widely used in clinical practice and its flavonoid compounds exhibit anti-CRC activity (Table 2), there is currently no large-scale clinical research supporting their efficacy. And most existing clinical studies used the combined administration of two or more flavonoid compounds. Systematic clinical evaluations of flavonoid interventions targeting for CRC pathological diversity and mutational heterogeneity are notably absent in current literature. Future research should also focus on the effective dosage and safety of individual flavonoid compounds from Herba *Patriniae*.

**TABLE 2:** Clinical studies on CRC prevention and treatment with the flavonoids.

Study design	Sample size(N)	Cancer type	Dosages/Drugs	Major outcome	Ref.
Prospective cohort comparison	N = 87	CRC/adenoma polypectomy	20 mg apigenin and 20 mg epigallocathechin-gallat	The combined recurrence rate for neoplasia was 7% (1 of 14) in the treated patients and 47% (7 of 15) in the controls	Hoensch et al. (2008)
Randomized, multicenter, nutritional intervention trial	N = 872	Colorectal adenoma	Dietary flavonols	Higher flavonol intakes and a reduction in serum IL-6 concentrations during the trial were associated both with decreased incidence of high-risk adenoma recurrence	Bobe et al. (2010)
Cohort study	N = 38,408 (middle-aged and elderly women)	All cancer risk	Dietary flavonols (quercetin、 kaempferol 、myricetin、apigenin、 luteolin)	There was also no significant association between intake of flavonoid-rich foods and the incidence of total and site-specific cancers	Wang et al. (2009)
Randomized, controlled, two part, single institution study	N = 180	Colon cancer	Oral rutin at 1 of 3 doses twice a day (arms III, IV, and V), oral quercetin at 1 of 3 doses twice a day (arms V, VI, and VII), or at 1 of 3 doses oral curcumin twice a day (arms VIII, IX, and X)	—	NCT00003365
Clinical research	N = 5	Familial adenomatous polyposis	Curcumin 480 mg and quercetin 20 mg orally 3 times a day	All 5 patients had a decreased polyp number and size from baseline after a mean of 6 months of treatment with curcumin and quercetin	Cruz-Correa et al. (2006)

## Mechanisms of action of flavonoids in their anticancer activity against CRC

The majority of colorectal cancer cases follow the classical adenoma-carcinoma sequence: normal colonic epithelium -adenomatous polyp–adenocarcinoma (Li et al., 2021). This carcinogenic progression begins with a mutation in the APC gene, followed by further mutations in KRAS, TP53, DCC and other critical genes (Schumacher et al., 2015). CRC pathogenesis involves the dysregulation of multiple pivotal signaling pathways: The Wnt/β-catenin pathway promotes tumor cell proliferation and differentiation, the PI3K/AKT pathway enhances cancer cell growth, survival, and metabolic adaptation, and the RAS/RAF/MEK/ERK cascade drives cell cycle progression and aberrant proliferation. In contrast, the transforming growth factor-β (TGF-β) pathway exhibits tumor-suppressive effects in the early stages, but promotes tumor invasion and metastasis through epithelial-mesenchymal transition (EMT) in the advanced stages of the disease. Current evidence demonstrates that flavonoids can effectively suppress CRC progression through multi-target mechanisms and the coordinated modulation of these oncogenic pathways (Li Q. et al., 2024).

### Inhibition of inflammation-associated tumors

**FIGURE 1:**
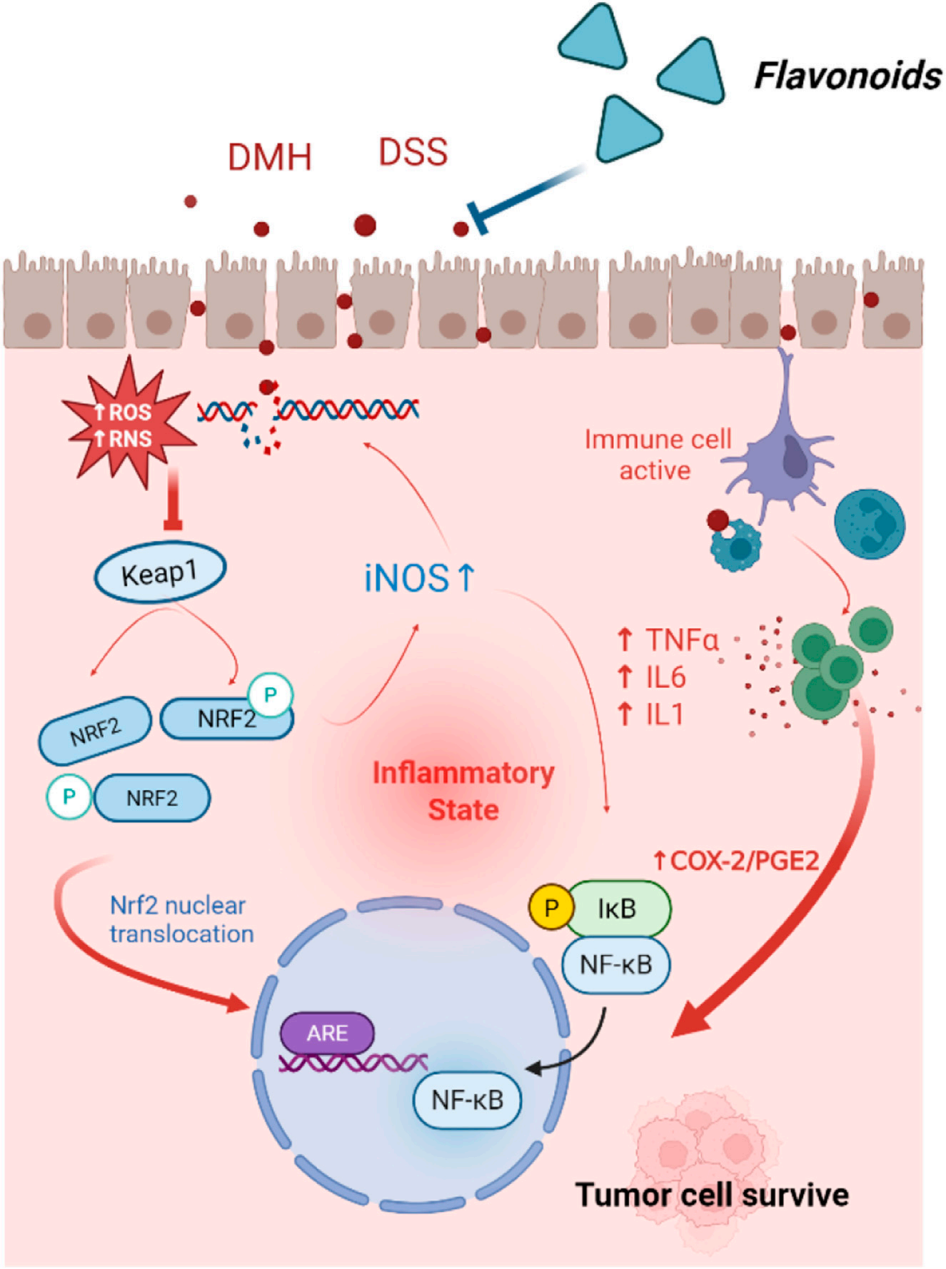
Mechanisms of Flavonoids inhibit inflammation-associated CRC.

Due to severe oxidative stress, reactive oxygen species (ROS) are released in chronic and sustained inflammation. Elevated levels of ROS can lead to DNA damage, thereby promoting the development of CRC. Interestingly, high levels of ROS in tumor cells present a potential therapeutic target (Lyons et al., 2023). The colon cancer inducer DMH has been shown to elevate ROS levels in the mouse models of CRC. Quercetin, administered at doses of 25 or 50 mg/kg can restore antioxidant response and mitigate membrane damage (Wang et al., 2009). The cytotoxic effects of quercetin on CRC cells are mediated through ROS-induced apoptosis and inhibition of cell survival pathways (Raja et al., 2017). Additionally, quercetin reverses DMH-induced oxidative stress and DNA damage by targeting the NRF2/Keap1signalling pathway. Compared to healthy individuals, patients with CRC exhibit more severe DNA damage in their lymphocytes (Figure 1) (Darband et al., 2020). *In vitro* studies have shown that quercetin (500 μM) reduces oxidative stress in lymphocytes of patients with CRC. Similarly, luteolin has been reported to suppress azoxymethane-induced CRC by activating the NRF2 signaling pathway (Pandurangan et al., 2014a). Elevated mitochondrial superoxide levels suggest that mitochondrial oxidative damage arises from an imbalance between anti-apoptotic and pro-apoptotic proteins, leading to dose-dependent cellular injury. Prolonged treatment with apigenin at growth-inhibitory doses was shown to induces persistent oxidative stress, ultimately triggering cellular senescence, a natural tumor suppression mechanism. In CRC cell lines HT-29 and HCT-15, apigenin has been shown to induce sustained oxidative stress at growth-inhibitory doses, leading to cellular senescence (Banerjee and Mandal, 2015).

During inflammation, immune cells are recruited to the intestines, where they release ROS and pro-inflammatory cytokines, such as TNF-α, IL-1, and IL-6. These factors can alter the intestinal microenvironment, promoting the growth and metastasis of CRC cells. Quercetin has been shown to inhibit the production of TNF-α and IL-6 (Han et al., 2016). Isoorientin, on the other hand, suppresses DSS-induced production of TNF-α, IL-β, and TNF-α induced activation of NF-κB by upregulating AHR, thereby protecting the integrity of the intestinal barrier (Mu et al., 2023).

Pro-inflammatory cytokines secreted by inflammatory cells can also activate the expression of various oncogenes and inhibit tumor suppressor genes. In the AOM/DSS-induced inflammation-associated tumor mouse model, quercetin inhibited the expression of COX-1, COX-2, and iNOS (Warren et al., 2009). Additionally, quercetin (IC50 = 10.5 μmol) effectively downregulated the transcription of COX-2 in human CRC DLD-1 cells (Mutoh et al., 2000).

Inflammation not only induces mutations through DNA damage but can also affect cancer-related genes via epigenetic modification, thereby silencing key tumor suppressor genes. Luteolin was shown to reduce methylation levels in the promoter region of Nrf2 and decreased protein levels and enzyme activities of DNMTs and HDACs in HCT116 cells. This finding suggests that luteolin may exert antitumor effects partly by epigenetically modulating the Nrf2 gene, thereby activating downstream antioxidant stress pathways via the Nrf2/ARE signaling pathway (Zuo et al., 2018). Following a thorough database screening, six key genes targeted by rutin in CRC were identified, including TP53, PCNA, CDK2, LDHA, CDKN1A and CCNB1. Molecular docking studies revealed that rutin exhibited a strong binding affinity for these targets. Following 48 h of rutin treatment in HT29 cells, the mRNA expression levels of the CRC target genes PCNA, CDK2, LDHA and CCNB1 decreased significantly. Conversely, TP53 and CDKN1A expression levels increased. Taken together, these results suggest that rutin treatment exerts regulatory effects in HT-29 cells and is involved in the ROS pathway (Gatasheh, 2024).

### Inhibition of CRC cell lines proliferation

#### PI3K/Akt/mTOR signaling

**FIGURE 2:**
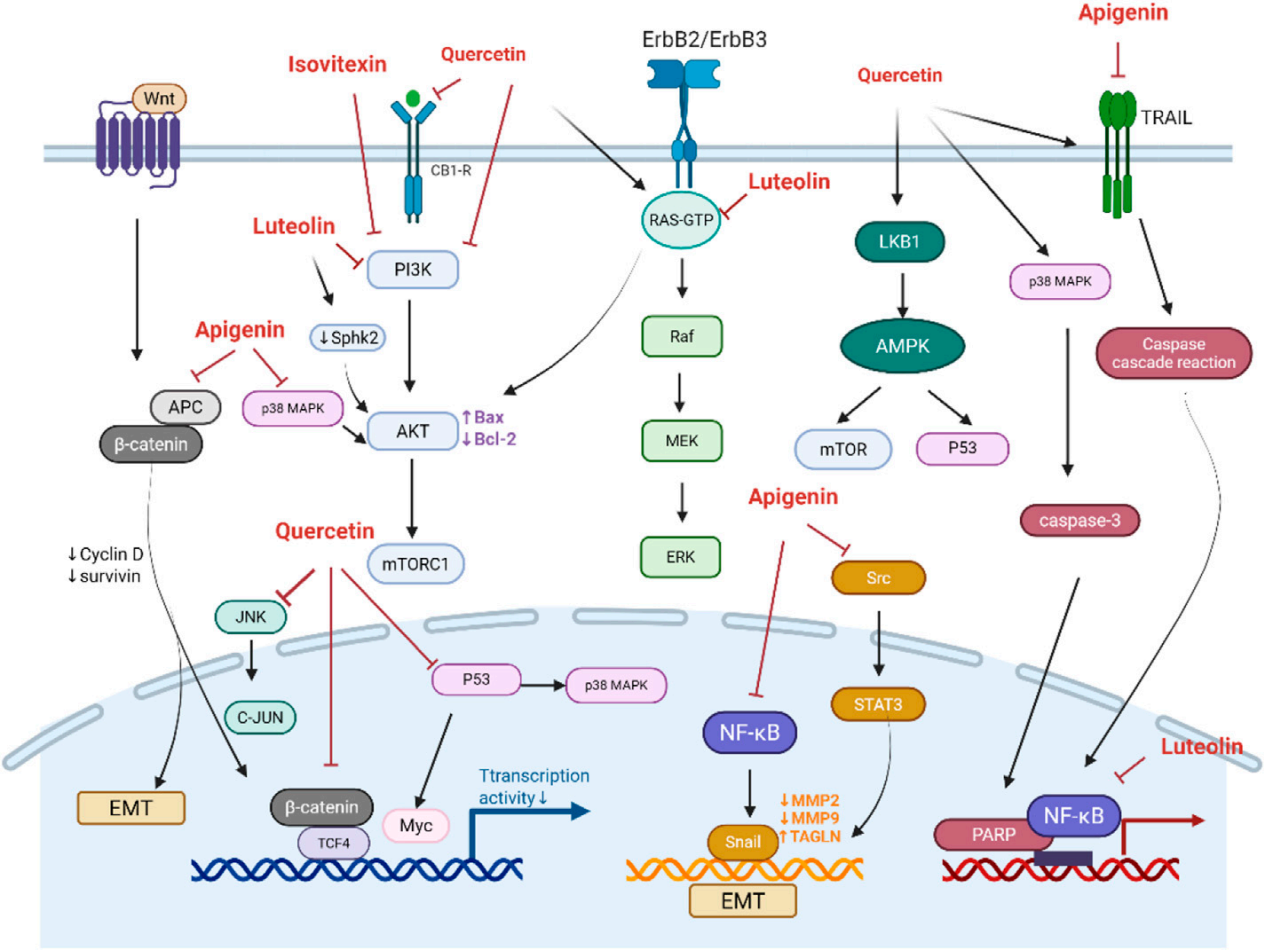
Potential mechanism of flavonoids against colorectal cancer.

The PI3K/Akt pathway is a critical signaling pathway in CRC development, which promotes cell survival and growth and cell cycle progression. Due to its estrogen-like structure, quercetin can bind to CB1-R, which is an estrogen-responsive receptor. This binding inhibits the PI3K/AKT/mTOR pathway in human colorectal adenocarcinoma cells (Caco2 and DLD-1) and activates the pro-apoptotic JNK/JUN pathway (Zuo et al., 2018). Quercetin also directly interacts with PI3K, reducing the expression of p-PI3K and p-AKT proteins and upregulating Bax and caspase-3 proteins. Consistently, quercetin inhibited cell proliferation and promoted the apoptosis of SW480 cells (Na et al., 2022). Additionally, quercetin downregulates the ErbB2/ErbB3 signaling pathway and the Akt pathway and lowered Bcl-2 levels, which suppressed the growth of HT29 and SW480 cells and induced their apoptosis (Kim et al., 2005). Luteolin exerts cytotoxic effects by inhibiting Akt activation and SphK2, thereby reducing S1P, an activator of Akt (Figure 2) (Kim et al., 2005).

#### Wnt/β-catenin signaling

In CRC, mutations in the APC gene or excessive activation of β-catenin can lead to its accumulation and nuclear translocation, which promotes the transcriptional activation of oncogenes, such as c-Myc and Cyclin D1, thereby accelerating cell proliferation. The interaction between β-catenin and TCF4 acts as a “molecular switch” in the Wnt signaling pathway. Treatment of SW480 cells with quercetin (160 μmol/L) for 24 h reduced the transcriptional activity of β-catenin/TCF by 18-fold, dose-dependently downregulated the transcription and protein expression of Cyclin D1 and survivin, inhibiting cell proliferation (Shan et al., 2009). Similarly, morusin inhibits Akt, which leads to an increased expression and activation of Gsk-3β. The activated Gsk-3β subsequently reduces the expression of β-catenin, resulting in a decrease in TCF4 expression. Consequently, morusin suppresses key downstream targets of the Wnt/β-catenin pathway (c-Myc and survivin) in a concentration-dependent manner (Zhou et al., 2021). Apigenin-induced dysfunction of APC is a key mechanism responsible for inducing cell cycle arrest in HT29-APC cells. Additionally, apigenin enhances APC expression and promotes apoptosis in wild-type APC cells (Figure 2) (Chung et al., 2007).

#### EGFR/ERK/MAPK signaling

The activation of EGFR triggers the Ras/Raf/MEK/ERK signaling cascade, which promotes cell proliferation, migration, and invasion. Luteolin was found to mediate the MAPK signaling pathway, thereby inhibit the proliferation of CRC cells (HCT116, HT29), and inducing cell cycle arrest, DNA damage, and apoptosis (Chung et al., 2007). A combination of apigenin (25 μmol) and chrysin (25 μmol) synergistically inhibited CRC cells growth and metastasis by suppressing the P38-MAPK/AKT pathway (Zhang et al., 2021). Quercetin inhibited the ERK/MAPK pathway and targets LKB1 to activate AMPK, thereby enhancing autophagy in radioresistant human CRC cells (HT500) (Russo et al., 2023). Quercetin also enhanced the expression of Sestrin 2 and p53, and activated AMPK/p38, AMPK-mTOR, and AMPK/COX-2 pathways (Lee et al., 2009; Kim et al., 2010; Kim G. T. et al., 2013; Kim et al., 2014).

Tumor cell growth is closely associated with cell cycle progression. Luteolin exhibited potent anti-proliferative properties in human CRC cells by promoting apoptosis, increasing the number of cells in the G1 phase, and reducing the number of cell in the S phase, while enhancing the proliferation of HF cells (Gennari et al., 2011). Furthermore, luteolin shifted oxaliplatin-induced G0/G1 arrest in HCT116 cells to apoptosis (Jang et al., 2019). Phytoestrogens mimic the effects of estradiol and induce apoptosis by interacting with ERα and ERβ. Quercetin modulates hormone receptor ESR2 and GPR30 related signaling pathways, arresting HT-29 cells in the G0/G1 phase, while fermented quercetin (FEQ) extract induces S phase arrest (García-Gutiérrez et al., 2023). The combination of ERβ ligands and tamoxifen (TMX) was found to induce tumor stasis in CRC cells (García-Gutiérrez et al., 2023). Moreover, apigenin reduces ER-mediated YAMC cell growth (Yang et al., 2015).

### Induction of apoptosis in CRC cells

#### Intrinsic Pathway

The intrinsic pathway activates cell death through the release of apoptosis-related factors from the mitochondria. Quercetin has been shown to activate p38 in DLD-1 cells, promoting caspase-3 activation, PARP cleavage, and cell death (Bulzomi et al., 2012). Additionally, quercetin mediates apoptosis in HT-29 cells by modulating Akt phosphorylation and promoting the degradation of CSN6 protein, which affects the expression levels of Myc, p53, Bcl-2, and Bax. This suggests that quercetin-induced apoptosis in HT-29 cells may involve the Akt-CSN6-Myc signaling axis (Yang et al., 2016). Apigenin induces apoptosis in CRC cells by simultaneously inhibiting Bcl-1and Mcl-1 via the STAT3 signaling pathway (Maeda et al., 2018).

#### Extrinsic Pathway

In the extrinsic pathway, FADD activates caspase-8, finally initiating apoptosis by activating caspase-3. Apigenin enhances the expression and phosphorylation of FADD, potentially leading to CRC cell apoptosis and inhibiting tumor growth (Wang et al., 2011). Tumor necrosis factor ligand superfamily member 10 (TRAIL) triggers apoptotic signaling by binding to its receptors DR4 (TRAIL-R1) and DR5 (TRAIL-R2). Quercetin facilitates the redistribution of DR4 and DR5 on the cell surface, enhancing TRAIL-induced caspase cascade (Psahoulia et al., 2007) and the NF-κB pathway to induce apoptosis (Zhang X. A. et al., 2015). A novel dual-targeting oncolytic adenovirus, combining complement CD55-TRAIL, synergistically suppresses tumor growth and induces CRC cell apoptosis both *in vitro* and *in vivo* (Xiao et al., 2017). Apigenin disrupts TRAIL resistance in HTLV-1-associated ATL by transcriptionally downregulating c-FLIP (a key inhibitor of death receptor signaling) and upregulating TRAIL-R2 (Ding et al., 2012).

#### JNK and p38 MAPK Pathways

KRAS-mutant cells are more sensitive to quercetin-induced apoptosis compared to wild-type cells (Xavier et al., 2009). Quercetin selectively activates the c-Jun N-terminal kinase (JNK) pathway in KRAS-mutant cells (Yang et al., 2019). By inhibiting NF-κB, luteolin was shown to enhance TNF-α-induced activation of JNK (Shi et al., 2004).

#### P53 Pathway, miRNA Regulation, and Others

Isovitexin induces apoptosis and cell cycle arrest via activating p53, thereby protecting against CRC (Li et al., 2023). Apigenin (Lee et al., 2014) and kaempferol (Choi et al., 2018) were shown to induce PARP cleavage and decrease the levels of caspases −8, −9, and −3, finally promoting apoptosis in CRC cells, however, the pro-apoptotic effects of kaempferol were reversed by ROS and p53 signaling.

miRNA-215-5p acts as a tumor suppressor, directly binding to and degrading the mRNA of E2F1 and E2F3, and inhibiting their protein synthesis. E2F1 and E2F3 play key roles in the G1/S phase of the cell cycle. Apigenin was found to downregulates their expression, resulting in cell cycle arrest and reduced cancer cell proliferation of CRC cells (Cheng et al., 2021). Kaempferol inhibits the nonoxidative pentose phosphate pathway (PPP), reducing ribose-5-phosphate (R5P) production and causing DNA damage. Mechanistically, kaempferol upregulates microRNA-195/497 (miR-195/497), which directly binds to the 3′-UTR of PFKFB4 mRNA to suppress PFKFB4 expression. This downregulation inhibits key nonoxidative PPP enzymes transketolase (TKT) and transaldolase (TALDO) (Wu et al., 2025). Apigenin also induces G2/M phase arrest in SW480 and Caco-2 cells (Wang et al., 2004). Concentration-dependent inhibition of HCT116 cell growth by apigenin leads to G2/M arrest, suppression of cyclin B1 and its activation partners cDC2 and CDC25C, and increased expression of the cell cycle inhibitors p53 and p53-dependent p21 (CIP1/WAF1).

Acacetin induces apoptosis in a caspase-independent manner by triggering mitochondrial ROS-mediated cell death through apoptosis-inducing factor in SW480 and HCT-116 CRC cells (Prasad et al., 2020). Luteolin regulates apoptosis signaling in BE CRC cells by downregulating the expression of calpain, UHRF1, and DNMT1 (Krifa et al., 2014). PKM2 is a key enzyme involved in the metabolic reprogramming of cancer cells, and studies have indicated that apigenin can target the K433 site of PKM2, inhibit glycolysis and suppress the proliferation of CRC cells and tumor progression both *in vitro* and *in vivo* (Shi et al., 2023).

### Inhibition of CRC cell invasion and metastasis

Approximately 90% of cancer-related deaths are attributed to distant metastasis. CRC is known for its high metastatic potential, making the inhibition of metastasis crucial for effective treatment. The mechanisms underlying metastasis are complex, with epithelial-mesenchymal transition (EMT) being a key process. Tumor cells need to undergo EMT and lose their epithelial characteristics to acquire invasive and migratory capabilities. Downregulation of E-cadherin and upregulation of N-cadherin are two hallmarks of EMT.

Quercetin has been identified as a promising therapeutic agent for the treatment of refractory cancers and preventing EMT-mediated metastasis. It modulates EMT markers such as E-cadherin, N-cadherin, β-catenin, and Snail, thereby inhibiting the migration and invasion of Caco-2 and CT26 cells (Han et al., 2016; Kee et al., 2016). TGF-β is a well-known inducer of EMT, and quercetin has been shown to reverse TGF-β1-induced morphological changes and EMT-like phenotypes in SW480 cells by inhibiting the expression of Twist1 (Feng J. et al., 2018). Similarly, luteolin suppresses EMT in CRC cells at the transcriptional level by downregulating CREB1 expression (Liu et al., 2017). Isovitexin reduces the levels of p-PI3K, p-Akt, p-mTOR, and Bcl-2 in tumor tissues, thereby inhibiting the migration, invasion, and EMT of cancer cells (Zhu et al., 2021). Additionally, isoorientin inhibits EMT and reversed cancer stem cell-like traits in oral squamous carcinoma by blocking the Wnt/β-catenin/STAT3 axis (Liu et al., 2021).

Apigenin also exhibited strong anti-EMT properties by inhibiting the Wnt/β-catenin (Xu et al., 2016) and NF-κB/Snail signaling pathways in human CRC cells (Tong et al., 2019). It hindered the migration, invasion, and metastasis of CRC cells through the NEDD9/Src/Akt cascade (Dai et al., 2016). Notably, apigenin upregulates cell surface protein CD26 and enhanced DPPIV activity in HT-29 and HRT-18 human CRC cells, further inhibiting tumor metastasis. The combination of apigenin with chemotherapeutic agents, such as irinotecan, 5-fluorouracil, and oxaliplatin, enhanced the CD26 for advanced CRC (Lefort and Blay, 2011). Pu et al. found that circ_0000345 promotes CRC metastasis by activating the JMJD2C/β-catenin pathway through miR-205-5p (Pu et al., 2024). In addition, they observed that kaempferol suppresses the expression of circ_0000345, effectively blocking JMJD2C/β-catenin signaling and inhibiting the lung metastasis of CRC.

MMPs are critical effectors in the EMT process. Quercetin and luteolin downregulate the expression of metastasis-related proteins MMP-2 and MMP-9 (Kim H. Y. et al., 2013; Han et al., 2016), as well as tissue inhibitors of metalloproteinases (TIMPs) (Pandurangan et al., 2014b; Kee et al., 2016). Apigenin upregulates TAGLN, which in turn downregulates MMP-9 expression and prevents cell proliferation and migration by reducing Akt phosphorylation at Ser473 and particularly at Thr308 (Chunhua et al., 2013).

### Enhancing cell sensitivity to drugs or radiation

Recently, natural compounds in cancer treatment has gained increasing attention in clinical practice. Studies have shown that flavonoids found in *Patrinia* scabiosaefolia, such as quercetin, luteolin, apigenin, and isoorientin, can enhance the cytotoxic effects of various chemotherapeutic agents on tumor cells, which may provide more options for CRC (Özerkan, 2023).

Cisplatin (CP) and oxaliplatin are the most commonly used platinum-based chemotherapeutics agents for treating CRC. However, their application is limited by their toxic effects on normal tissues and drug resistance. Quercetin, when combined with CP, has been shown to reduce ACF while enhancing the efficacy of CP and mitigating its nephrotoxicity (Li et al., 2016). Apigenin inhibits tumorigenesis in cisplatin-resistant CRC cells both *in vitro* and *in vivo* by inducing autophagy, and programmed cell death and targeting the mTOR/PI3K/Akt signaling pathway (Chen et al., 2019). Additionally, isoorientin activates the SIRT1/SIRT6/Nrf2 pathway to reduce oxidative stress and apoptosis, thereby alleviating cisplatin-induced nephrotoxicity (Fan et al., 2020). Kaempferol has been shown to inhibit AP-1 transactivation, thereby enhancing the inhibitory effect of oxaliplatin on HCT116 and HT29 cells (Park et al., 2021).

P-glycoprotein (P-gp)-mediated multidrug resistance (MDR) presents a significant challenge to successful chemotherapy. Studies have indicated that quercetin enhances the antiproliferative effects of doxorubicin on P-gp-overexpressing SW620/Ad300 cells by inhibiting ATP-driven transport activity, which increases the intracellular accumulation of doxorubicin. The study also suggests that quercetin may reverse MDR by disrupting D-glutamine and D-glutamate metabolism (Zhou et al., 2020). Moreover, isoorientin reduces doxorubicin-induced cardiotoxicity by activating MAPK, Akt, and caspase-dependent pathways (Li S. et al., 2022).

OCTN2, a member of the solute carrier superfamily and a key determinant for oxaliplatin uptake. Luteolin enhanced oxaliplatin absorption and intracellular accumulation through the PPARγ/OCTN2 pathway, thereby sensitizing SW480 cells to oxaliplatin (Qu et al., 2014). By inhibiting AMPK, luteolin synergistically improves the antitumor efficacy of oxaliplatin in CRC (Jang et al., 2022), and sensitized oxaliplatin-resistant CRC cells to chemotherapy by suppressing the Nrf2 pathway (Chian et al., 2014). Combined treatment with quercetin and oxaliplatin synergistically inhibited glutathione reductase activity, increasing ROS production, and induced glutathione depletion, thereby enhancing oxaliplatin sensitivity in CRC cells (Lee et al., 2023).

5-Fluorouracil (5-FU) is a commonly administered chemotherapeutic agent for treating CRC, but its efficacy is often limited by acquired resistance in advanced stages. Thymidylate synthase (TS), the target protein of 5-FU, is upregulated in CRC and contributes to 5-FU resistance. Apigenin has been shown to enhance the inhibitory effect of 5-FU on cell viability, induce apoptosis in HCT116 cells, and promote cell cycle arrest, likely by inhibiting TS expression (Yang et al., 2021). Likewise, kaempferol synergistically enhanced the effects of 5-FU by suppressing TS expression and inhibiting p-Akt (Li et al., 2019). Additionally, studies have shown that miR-27a can promotes the proliferation of CRC cells through the Wnt/β-catenin pathway. A combination of quercetin and 5-FU exhibitsed stronger cytotoxicity than 5-FU alone. Besides, quercetin enhanced 5-FU sensitivity in CRC by inhibiting the miR-27a/Wnt/β-catenin signaling axis (Terana et al., 2022). Furthermore, quercetin downregulates 5-FU-induced TS levels, upregulates p53 expression, induced ROS production and Ca^2+^ dysregulation through non-5-FU-dependent pathways in CRC cells. Moreover, quercetin enhanced sensitivity to 5-FU in mice with colitis-associated CRC (Yang et al., 2022). Kaempferol reduced glucose uptake and lactate production in drug-resistant CRC cells, and increased the expression of microRNA-326 (miR-326), which targets PKM2 and inhibits glycolysis, thereby reversing 5-FU resistance (Wu et al., 2022).

Luteolin has also been shown to reverse inflammation and oxidative imbalance induced by irinotecan via PPARγ-dependent downregulation of IL-1β and iNOS (Boeing et al., 2022). Treatment with quercetin enhanced the expression of NKG2D ligands on cancer cells, making them more susceptible to NK cell-mediated cytotoxicity (Bae et al., 2012). Furthermore, the combination of quercetin and ionizing radiation (IR) targeted colon cancer stem cells and inhibits Notch-1 signaling (Li et al., 2020). Both apigenin and luteolin exhibited efficacy in targeting the PI3K/Akt/mTOR axis, making them as promising non-toxic alternatives to synthetic chemical drugs used for the treatment of CRC (Sain et al., 2022).

## Interaction of flavonoids with gut microbiota

Gut microbiota plays a crucial role in the progression of CRC, and targeting gut microbiota is an important pharmacological strategy for treating CRC (Jia et al., 2024). Flavonoids are not easily absorbed by the human body after oral intake. Most flavonoids are metabolized by gut microbiota into smaller, more absorbable active metabolites, thus enhancing their bioactivity.

**FIGURE 3:**
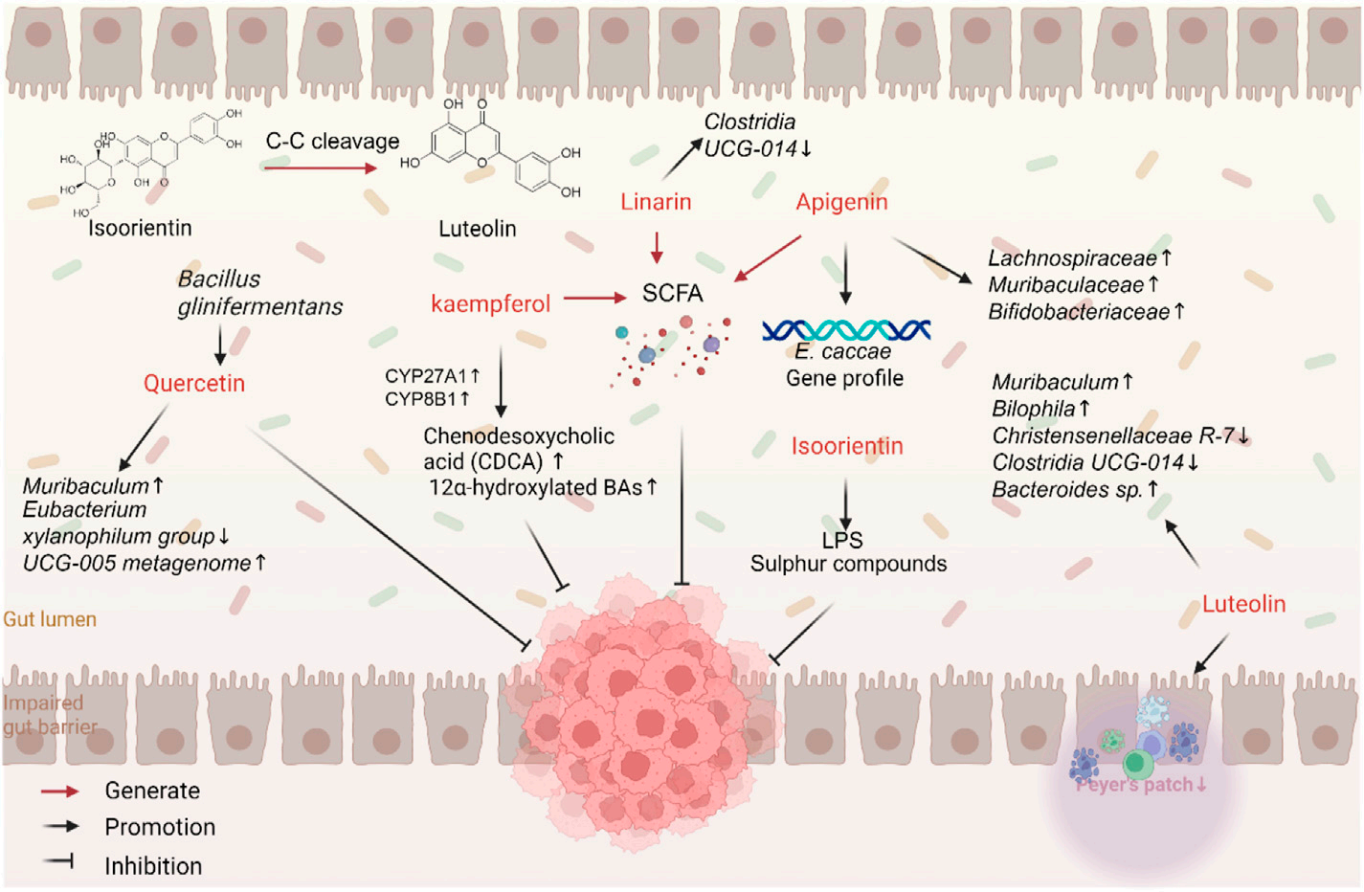
Flavonoids interact with gut microbiota against CRC.

Gut microbiota can transform flavonoids by altering their chemical structure and biological functions, thereby enhancing their anticancer potential (Figure 3). Specific bacterial strains, such as *Bifidobacterium* and *Lactobacillus*, play essential roles in this metabolic process. Flavonoids mainly exist as glycosides, with only 5%–10% being directly absorbed, resulting in low bioavailability. The majority of flavonoids are metabolized by gut microbiota into smaller molecules, such as phenolic acids, which increases the abundance of beneficial bacteria and stimulates the production of short-chain fatty acids (SCFAs) (Gonzales et al., 2015; Feng X. et al., 2018). C-glycoside flavonoids are highly metabolized in the gut. For example, enzymes expressed by Caco-2 cells can cleave C-C bonds *in vitro*, breaking the bonds between sugar and aglycone residues in orientin and isoorientin to produce glucuronidated or sulfated derivatives of luteolin and apigenin (Dihal et al., 2006; Tremmel et al., 2021). Studies have shown that *Bacillus glycinifermentans*, *Flavonifractor plautii*, *Bacteroides eggerthii*, *Olsenella scatoligenes*, and *Eubacterium eligens* can degrade quercetin. *B. glycinifermentans* produces metabolites like 2,4,6-THBA and 3,4-DHBA, which inhibit the proliferation of CRC cells (Sankaranarayanan et al., 2021). 3,4-Dihydroxyphenylacetic acid, a microbial metabolite derived from quercetin, was shown to strongly inhibit the CRC-promoting properties of heme chloride than quercetin itself and prevent heme-induced malignant transformation of colonic epithelial cells and mitochondrial dysfunction (Catalán et al., 2020). The metabolism of flavonoids is a complex process involving various gut microbiota, which can sustain or even enhance their anticancer effects (Zhang et al., 2014; Cattivelli et al., 2023).

On the other hand, flavonoids can regulate the composition of gut microbiota by promoting the growth of beneficial bacteria and inhibiting the proliferation of pathogenic and pro-inflammatory bacteria. In a mouse model of cancer, luteolin has been shown to significantly reduce the abundance of disease-associated or inflammation-associated genera, such as *Clostridium* UCG-014 and *Turicibacter*, while increasing the abundance of *Muribaculaceae*, a health-promoting genus. This finding supports its antitumor effect through microbiota modulation (Pérez-Valero et al., 2024). Similarly, apigenin can mitigate gut dysbiosis by increasing the abundance of beneficial bacteria, such as *Lachnospiraceae*, *Muribaculaceae*, and *Bifidobacterium* (Rithidech et al., 2024), and affecting the growth and gene expression of *Enterococcus* (Wang et al., 2017). Interestingly, the anticancer effects of apigenin relied on gut microbiota (Bian et al., 2020). Isoorientin has significant advantages in preventing colon damage and gut dysbiosis induced by benzo [a]pyrene (BaP). Isoorientin was found to changes the abundance of gut microbiota, especially *Feacalibaculum*, *Lactobacillus*, *Acetobacter*, *Desulfovibrio*, and *Alistipes,* after exposure to BaP. Isoorientin also improves metabolic disorders of gut microbiota after exposure to BaP. Particularly, it improved perturbations in pathways involving LPS and sulfur compounds (He et al., 2019). Linarin reversed DSS-induced gut microbiota dysbiosis, affecting the abundance of different genera, such as *Alistipes*, *Rikenella*, and *Clostridia* UCG-014_norank. It also increased the abundance of SCFA-producing bacteria, like *Lactobacillus*, *Roseburia*, *Parabacteroides*, and *Blautia* (Jin et al., 2022).

Dysbiosis of gut microbiota can affect the production of metabolites, such as bile acids (BAs), LPS, choline, and SCFAs. Quercetin can modulate the composition of gut microbiota, improve the integrity of the intestinal barrier (Mohammadhasani et al., 2024), and reduce serum levels of hippuric acid (HA), a polyphenol-derived metabolite, observed in patients with Crohn’s disease. HA levels in the gut were found to be positively correlated with polyphenol intake, the abundance of flavonoid-degrading bacteria, and SCFA production (Xiang et al., 2024). Kaempferol can increase the expression of enzymes such as sterol CYP27A1 and sterol CYP8B1, thereby increasing the decreased levels of CDCA and 12α-hydroxylated bile acids and upregulating FXR expression. This finding suggests that kaempferol can downregulate secondary bile acid synthesis pathways, increase G-protein-coupled receptor activity, and decrease NLRs activity, thereby influencing cell differentiation, proliferation, survival, and apoptosis (Li X. et al., 2022). However, further exploration is needed to fully explore the mechanisms through which flavonoids interact with gut microbiota and their metabolites.

Through their bidirectional interaction with gut microbiota, flavonoids promote gut health and inhibit the development of CRC. They are transformed into more active metabolites and modulate the composition of gut microbiota, resulting in anti-inflammatory, antioxidant, and immunomodulatory anticancer effects in cancer. This interaction provides new insights and research directions for the prevention and treatment of CRC.

## Construction and application of drug delivery systems

Flavonoids have shown significant potential in the prevention and treatment of CRC. However, their poor water solubility limits both their bioavailability and therapeutic efficacy. Additionally, the metabolism of flavonoids can further reduce their bioavailability and diminish their therapeutic potential. Nanomaterials offer unique advantages in targeted drug delivery. They can protect drugs against degradation, enhance drug solubility, reduce toxicity, and enhance pharmacodynamics and pharmacokinetics. The application of nano-engineered flavonoids not only improves the antitumor effects of chemotherapy drugs but also reduces their systemic toxicity (Table 3).

**TABLE 3:** Flavonoid nanostructures in the treatment of colorectal cancer.

Flavonoids	Classification of Materials	Cell Line	Encapsulation efficiency	Major Outcome
Quercetin	Liposome	HCT-116 p53^+/+^ cells	42%	Loaded liposomes exhibit higher antitumor effects compared to free quercetin
Quercetin	Shellac nanocapsules	HT-29、HCT-116	80%	48% of encapsulated curcumin and quercetin were bioaccessible, with increased cytotoxicity
Apigenin	Whey protein isolate	HT-29、HCT-116、*vivo*	98.15%	Enhancement of sub-G1 cell cycle arrest, possible induction of apoptosis, and improved bioavailability of apigenin in mouse serum and colonic mucosa
Apigenin	Aptamer-conjugated nanoparticle	HCT-116	17.5% ± 1.3%	Enhanced therapeutic efficacy to colorectal cancer cells
Apigenin	Gold nanoparticles	CT26	—	In the apoptosis triggered cell death in photothermal treatment
Apigenin	Liposome	HCT-15 and HT-29 、*vivo*	89.98% ± 2.31%	Stronger inhibition of angiogenesis, anti-proliferative, pro-apoptotic; *in vivo* anti-tumorigenesis

Quercetin, for example, exhibits low solubility in neutral and hydrophilic fluids. Encapsulation with soybean polysaccharide and chitosan has been shown to enhance the stability and solubility of quercetin-loaded flavonoids. Multiple independent studies have confirmed that quercetin-nanocarriers can improve their pharmacokinetics and absorption, thereby enhancing their anticancer efficacy against CRC. For instance, encapsulating quercetin in nanocapsules potentiated its antioxidant and cytotoxic activity in HT-29 and HCT116 cells (Jain et al., 2023). Additionally, the co-administration of quercetin with nanosynthesized drugs revealed improved antitumor efficacy (Colpan and Erdemir, 2021).

Flavonoids also exhibit antioxidant properties by chelating transition metals involved in free radical generation. Metal-flavonoid complexes act as more potent free radical scavengers than isolated flavonoids. For example, a quercetin-ruthenium complex reduced HT29 cell proliferation and induce tumor cell apoptosis by upregulating p53 and Bax and downregulating Bcl2 expression. The radical scavenging ability and antimicrobial activity of isoorientin-Zn were significantly stronger than those of isoorientin alone (Wang et al., 2021). Furthermore, the use of microencapsulated *Bifidobacterium bifidum* and *Lactobacillus gasseri*, either alone or in combination with quercetin, inhibited of CRC progression in Apc^Min/+^ mice (Benito et al., 2021).

Apigenin nanoformulations have been applied for targeted tumor cell treatment. Nano-encapsulation of apigenin enhanced cellular uptake, pro-apoptotic effects, and bioavailability in mouse blood and colonic mucosa (Dutta et al., 2018; Amini et al., 2021; Hong et al., 2022). Liposomal nanocarriers of apigenin exhibited anti-angiogenic properties, reduced cell proliferation, and increased cell apoptosis. Preclinical trials using these formulations in nude mouse xenograft models revealed enhanced antitumor effects (Sen et al., 2019). Luteolin has low oral bioavailability due to its poor water solubility, which maks its intravenous or intraperitoneal administration impossible. Nanoformulations help overcome these challenges, improving the anticancer efficacy of luteolin.

## Current challenges and future prospects

The flavonoids found in Herba *Patriniae* possess extensive anti-tumor properties, and their therapeutic effects in CRC have gained significant attention. These flavonoids show great promise for the development of new therapeutic agents.

**FIGURE 4:**
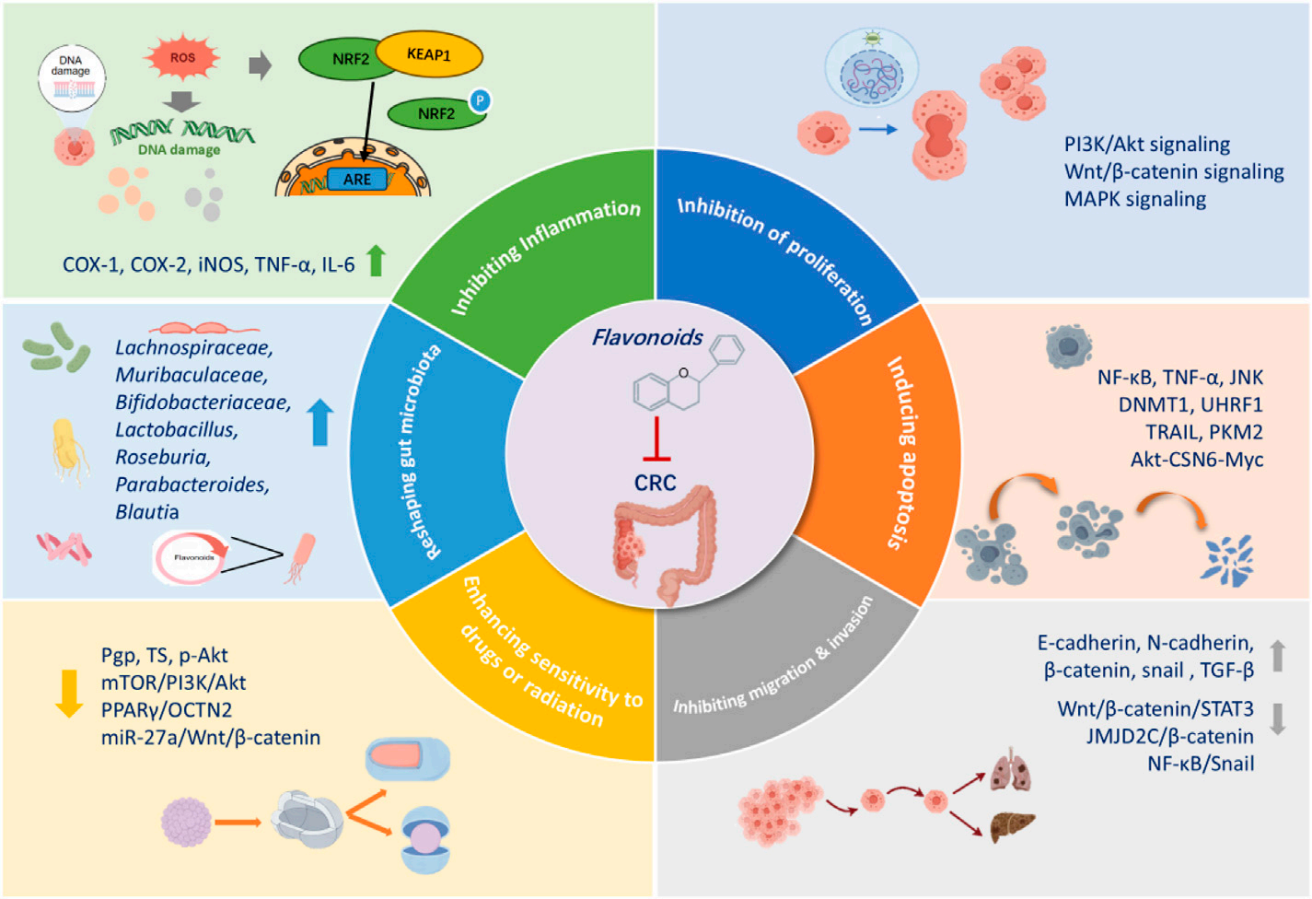
Mechanisms of action of flavonoids from patriniae in the anticancer activity against colorectol cancer.

Regarding pharmacological mechanisms, current studies suggest that flavonoids exert anti-tumor effects through various pathways (Figure 4). For instance, it was shown that flavonoids can reduce ACF, regulate cell cycle, inhibit the growth and proliferation of CRC cells, promote apoptosis, and suppress EMT, reversing CRC resistance, and modulate the composition of gut microbiota. A growing number of studies have focused on the crucial role of the tumor microenvironment (TME) in cancer progression. Recent findings unveiled that flavonoids from Herba *Patriniae* can affect the TME, including macrophage phenotypes. However, most studies have primarily observed the effects of flavonoids on stromal cells, and a deeper understanding of their mechanisms of action is still needed.

Well-designed randomized controlled trials are needed to confirm the safety and efficacy of flavonoid-based therapies. Although Herba *Patriniae* is widely consumed in tropical and subtropical regions, few clinical trials have administered *Herba Patriniae* and its flavonoids to patients with CRC. Such studies should evaluate the safety and tolerability of Herba *Patriniae*, and determine the maximum tolerated dose (MTD) and dose-limiting toxicity (DLT). High-quality Phase II–III clinical studies can provide higher-level evidence regarding efficacy. In addition, large-scale longitudinal interventional studies are important for understanding the metabolic variability of flavonoids for developing personalized therapeutic strategies based on gut microbiota.

Low bioavailability is also a major obstacle limiting the clinical application of flavonoids in Herba *Patriniae*. However, the development of novel nanoformulations holds promise for improving drug delivery, targeting specific tissues, and enhancing the bioavailability of these compounds. Modifying flavonoids based on their mechanisms of action can also advance the therapeutic use of natural plant compounds in the treatment of CRC.

Although some progress has been made in understanding the interaction between gut microbiota and flavonoid metabolites, there are many unknown aspects. The ability of gut microbes to metabolize flavonoid and the specific metabolites involved in the pathogenesis of CRC are still poorly understood, with limited data from clinical samples. More evidence from sterile mouse models and clinical studies is needed to address these gaps and explore the specific mechanism.

## Glossary

ACF: Abnormal crypt foci
AHR: Aryl hydrocarbon receptor
AKT: protein kinase B or PKB
AOM: Azoxymethane
APC: Adenomatous Polyposis Coli gene
AP-1: Activator protein-1
ARE: AU-rich element
ATL: Adult T-cell leukemia/lymphoma
Bax: Bcl-2-associated X protein
Bcl-2: B-cell lymphoma 2
CAC: Colitis-associated cancer
CA125: Cancer antigen 125
CB1-R: Cannabinoid receptor 1
CDCA: Chenodeoxycholic acid
cDC2: Type 2 conventional dendritic cell
CDC25C: Cell division cycle 25C
CDK6: Cyclin-dependent kinase 6
CD55: Decay-accelerating factor,DAF
c-FLIP: Cellular FLICE (FADD-like IL-1β-converting enzyme)-inhibitory protein
COX-1: Cyclooxygenase-1
COX-2: Cyclooxygenase-2
CP: Cisplatin
CRC: CRC
CREB1: cyclic-AMP response binding protein 1
CSCs: Cancer stem cells
CSN6: Constitutive photomorphogenesis 9 signalosome subunit 6
c-Myc: Myc proto-oncogene protein
CCND1: Cyclin D1
CYP8B1: Sterol 12-alpha-hydroxylase
CYP27A1: Cytochrome P450 Family 27 Subfamily A Member 1
DPPIV: Dipeptidyl peptidase IV
TRAIL-R1: TNF-related apoptosis-inducing ligand receptor 1,DR4
TRAIL-R2: TNF-related apoptosis-inducing ligand receptor 2,DR5
DSS: Dextran sulfate sodium
DMH: 1,2-dimethylhydrazine
DNA: DeoxyriboNucleic Acid
DNMTs: DNA methyltransferases
DNMT1: DNA methyltransferase1
EGFR: Epidermal Growth Factor Receptor
EMT: Epithelial-mesenchymal transition
ERα: Estrogen receptor α
ERβ: Estrogen receptor β,ESR2
ErbB2: Human epidermal growth factor receptor 2
ErbB3: Human epidermal growth factor receptor 3
ERK: Extracellular regulated protein kinases
E2F1: E2F Transcription Factor 1
E2F3: E2F Transcription Factor 3
FADD: Fas-associated protein with death domain
FAP: Familial adenomatous polyposis
FEQ: Fermented quercetin
FXR: Farnesoid X Receptor
GPR30: G protein-couple receptor 30,GPER
HDACs: Histone deacetylases
HTLV-1: Human T-lymphotropic virus 1
IBD: Inflammatory bowel disease
IL-1β: Interleukin-1 beta
IL-6: Interleukin-6
IL-10: Interleukin-10
iNOS: Inducible nitric oxide synthase
JMJD2C: Jumonji domain 2c
JNK: c-Jun N-terminal kinase
JUN: Jun proto-oncogen
Ki67: marker of proliferation
KRAS: Kirsten rat sarcoma viral oncogene
LGR5: Leucine-rich repeat-containing G protein-coupled receptor 5
LKB1: Liver kinase B
LPS: lipopolysaccharides
MAPK: Mitogen-activated protein kinase
MDR: Multidrug resistance
MEK: Mitogen-activated protein kinase kinase, MAPKK
mTOR: Mammalian target of rapamycin
MMP: Matrix metalloproteinases
NEDD9: Neural Precursor Cell Expressed, Developmentally Downregulated 9
NLRs: Nucleotide-binding and oligomerization domain-like receptors
NF-κB: Nuclear factor kappa-B
NK: Natural killer
NKG2D: Natural killer group 2D
NRF2: Nuclear factor erythroid 2-related factor 2
OCTN2: Organic cation/carnitine transporter 2
PARP: Poly ADP-ribose polymerase
p-Akt: Phospho-protein kinase B
P-gp: P-glycoprotein
PI3K: Phosphatidylinositol 3-kinase
p-PI3K: Phospho- Phosphatidylinositol 3-kinase
PKM2: Pyruvate kinase M2
PPARγ: Peroxisome proliferators-activated receptorsγ
Ras: Rat sarcoma
Raf: Raf-1 proto-Oncogene
ROS: Reactive oxygen species
SCFAs: Short-chain fatty acids
SIRT1: Silent information regulator 1
SIRT6: Silent information regulator 6
SphK2: sphingosine kinase2
SRC: SRC Proto-Oncogene
STAT3: Signal transducer and activator of transcription 3
S1P: Sphingosine-1-phosphate
TAGLN: Transgelin
TCF4: T-cell factor 4
TIMPs: Tissue inhibitors of metalloproteinases
TNBS: 2,4,6-trinitrobenzenesulfonic acid sol
TNF-α: Tumor necrosis factor-α
TRAIL: TNF Superfamily Member 10, TNFSF1O
TS: Thymidylate synthase
UHRF1: Ubiquitin-like with PHD and RING finger domains 1
Wnt: Wingless/Integrated
